# Clicker Training in Minipigs to Reduce Stress during Blood Collection—An Example of Applied Refinement

**DOI:** 10.3390/ani14192819

**Published:** 2024-09-30

**Authors:** Delia Fiderer, Christa Thoene-Reineke, Mechthild Wiegard

**Affiliations:** Institute of Animal Welfare, Animal Behavior and Laboratory Animal Science, School of Veterinary Medicine, Freie Universitaet Berlin, Koenigsweg 67, 14163 Berlin, Germany; thoene-reineke.christa@fu-berlin.de (C.T.-R.); mechthild.wiegard@fu-berlin.de (M.W.)

**Keywords:** animal welfare, positive reinforcement, refinement, stress reduction, cortisol, heartrate, minipigs, clicker training

## Abstract

**Simple Summary:**

In order to take blood from pigs or minipigs, they are often secured using a maxillary sling. The animals express strong vocalisations, and the procedure subjectively appears to be stressful for the animals. In the present study, 12 Ellegaard minipigs were operantly conditioned to tolerate blood sampling from the neck without restraint by using positive reinforcement. The aim was to initially quantify the extent of stress during blood sampling; to prove whether minipigs can be trained to tolerate aversive stimuli, such as the insertion of a cannula without fixation; and to prove whether stress can be reduced through training. After an initial blood sample with fixation in the maxillary sling, the animals were trained for 3 weeks, and then blood was taken again, but without fixation. Blood and saliva samples were taken to determine cortisol concentration. Heart rate was measured before and during blood sampling. The results showed no difference in the cortisol concentration in serum between trained and untrained animals, while it was significantly lower in saliva after blood sampling in the trained animals, as was the heart rate. The training proved to be a suitable method to reduce stress when taking blood from pigs.

**Abstract:**

Pigs (and minipigs) are often restrained with a maxillary sling for blood collection. They mainly produce strong vocalisations and show resistance to the procedure, which subjectively appears to be stressful for the animals. The present study investigated whether minipigs can be trained to tolerate aversive stimuli and whether training can reduce stress during blood collection. Blood was taken from 12 Ellegaard minipigs with fixation; thereafter, the animals were trained for 3 weeks using clicker training. Then, blood was taken again, but without fixation. Before and after each blood sample, saliva samples were taken. The cortisol concentration was determined using ELISAs. Serum cortisol was not significantly different before and after training (paired-sample *t*-test, t (9) = 2.052, *p* = 0.07). However, salivary cortisol was significantly lower after training (ANOVA (analysis of variance), *p*-value < 0.001, F-value 6.181). In addition, trained minipigs showed a significantly lower heart rate after blood sampling (paired-sample *t*-test, t (11) = 4.678, *p* = 0.001) as well as significantly lower heart rate variability (t (11) = 3.704, *p* = 0.003) compared to before training. The minipigs could be trained to tolerate aversive stimuli. This contributed to stress reduction when taking blood samples.

## 1. Introduction

Pigs are frequently enrolled in animal studies, and minipigs in particular have been extensively employed in biomedical research in recent years, providing a promising alternative to traditional animal models [1,2]. Minipigs have become increasingly valuable due to their anatomical and physiological similarities to humans [1,2]. These similarities extend to various organ systems, including the skin, cardiovascular, gastrointestinal, and urinary systems [1,3]; at the same time, minipigs offer advantages over traditional non-rodent models, such as dogs or monkeys, due to their smaller size, slower growth, ease of handling, and controlled genotype [1]. Recent advances in genetic engineering have enabled the creation of tailored porcine models for human diseases, making them valuable in preclinical studies and drug development [2,3]. Pigs are used in various research fields, including reproductive physiology, xenotransplantation, skin physiology, and neurotransplantation [4].

This study included 12 minipigs, subjected to blood sampling either performed the conventional way (with fixation in a maxillary sling (snout snare)) or following clicker training (to tolerate blood sampling without fixation). Blood samples drawn by both methods were compared. In addition, measurements of the heart rate (HR) and its variability during samplings were analysed, and as blood samples were already available, measurements of salivary and serum cortisol levels before, during, and after blood sampling were also performed.

Blood sampling in pigs is a standard procedure applied in research as well as conventional animal husbandry, with various techniques developed to minimise stress and improve efficiency. Jugular catheterisation offers a rapid, non-surgical method for repeated blood sampling in pigs of different sizes [5]. This approach can be performed quickly within pens and has no implications for either research or pharmaceutical testing. Alternative methods include a non-restrictive technique allowing sampling of grouped, unrestrained pigs [6] and a long-term catheterisation method for piglets that prevents catheter dislodgement during growth [7]. While blood sampling in minipigs can be challenging due to deeper blood vessels, proper training and the use of vascular catheters can minimise distress [8]. These techniques aim to reduce stress-induced physiological changes, as conventional venipuncture and restraint have been shown to elevate cortisol, glucose, and other parameters [7,8].

The standard method for taking blood from an adult pig or minipig is the puncture of the cranial vena cava or the external jugular vein with the animal standing fixed in a maxillary sling [9]. However, research indicates that blood sampling in pigs using traditional restraint methods can induce significant stress responses. Brenner and Gürtler observed increases in lactate, glucose, haemoglobin, and haematocrit levels following restraint [10]. Similarly, Marchant-Forde et al. found that manual sampling with physical restraint resulted in higher cortisol and noradrenaline concentrations compared to automated sampling methods [11]. Schrader and Todt demonstrated correlations between stress hormone levels and specific vocalisation patterns, with increased “squeal-grunts” indicating higher adrenaline levels and decreased “grunts” associated with elevated cortisol [12]. To minimise stress during blood sampling, Langner et al. developed a non-restrictive method for maintaining grouped swine [6]. These findings collectively suggest that alternative, less invasive techniques may be preferable for obtaining accurate physiological samples in pigs without inducing stress related artefacts.

When carrying out animal experiments, pain and stress caused to the animals must be limited to the indispensable minimum (Directive 63/2010/EU, German Animal Welfare Act). This implies that unnecessary impairments of animal wellbeing must be avoided, since they are ethically not justifiable. The 3R principle includes the improvement and refinement of procedures as an essential component [13]. According to Russel and Burch, refinement means that processes and methods in animal experiments should be enhanced with the aim of reducing the stress that the animals experience during the experiment to the minimum [13,14]. This aim can be reached by either improving the technical conduct of a certain procedure or improving the habituation or training of the animals in order to be better prepared for the planned interventions. This article will focus on the latter aspect.

The first aim of the present study was to determine the extent of stress during blood collection and to determine its root cause by distinguishing between fixation using a maxillary sling and puncture with a cannula in the neck region. 

Secondly, this study evaluated clicker training as an option for a refinement strategy in order to optimise procedures and reduce the stress of the animals [15,16,17]. A detailed training plan with individual training steps adaptable to the respective animals’ learning ability, willingness, and training success was set up to investigate whether tolerance of aversive stimuli such as the insertion of a cannula (and the subsequent blood collection) without fixation can be trained and whether this training could contribute to stress reduction and therefore be a means of applied refinement.

Clicker training comprised conditioning the pigs to the clicker sound as a conditioned trigger and secondary reinforcer and shaping behaviour by positive reinforcement—specifically, making the occurrence of a wanted behaviour that is rewarded by a primary reinforcer (e.g., food rewards, cuddling) become more probable [9]. Important preconditions for successful training are the identification of a powerful primary reinforcer and the precise timing of the clicker signal. 

In order to measure stress in pigs, it is possible to determine various biomarkers and changes in the body or behaviour that change due to the activation of the sympatho-adrenomedullary axis and the hypothalamic–pituitary–adrenal axis during stress [18]. In order to obtain measurable stress parameters during the course of the blood sampling, these were chosen on the basis of being non-invasive in order to avoid repeated invasive sampling and the risk of false results. 

Stress leads to adrenal activation with the release of glucocorticoids and catecholamines. In contrast to catecholamines, cortisol, as a representative of the glucocorticoids, can be determined non-invasively in saliva and is suitable for determining acute stress. An increase in cortisol in the blood (serum or plasma) occurs within one minute of exposure to stress, and the increase in saliva occurs with a slight delay of up to 5 min [9]. Salivary cortisol values correlate with serum cortisol values. As blood samples were available due to the original set-up of the experiment, there was an opportunity to additionally determine serum cortisol levels. 

The non-invasively measurable stress parameters also include HR, which increases due to sympathetic activation during stress [19,20]. Heart rate variability (HRV) has emerged as a promising non-invasive technique for assessing stress and welfare in farm animals, particularly pigs [21]. Studies have shown that HRV can effectively indicate stress responses in pigs during various situations, such as acute heat episodes [22], social stress [23], and pain from castration [22]. Both linear and non-linear HRV measures have been used to evaluate autonomic nervous system function and sympathovagal balance. Non-linear measures, including sample entropy and percentage determinism, have shown potential in complementing traditional linear measures for improved HRV interpretation [22]. Research has demonstrated that stressed pigs exhibit changes in HRV parameters, such as an increased low frequency/high frequency ratio and decreased sample entropy, compared to unstressed controls [22]. These findings suggest that HRV analysis can contribute significantly to understanding stress responses and welfare status in pigs.

In the present study, the HR was measured non-invasively using HR sensors on chest straps (as successfully used in previous studies [24,25]). Assessment of HRV in pigs can be performed if data over a period of time are available. Previous studies found that chronic distress caused by a change in housing from pair housing to individual housing leads to reduced HRV. This condition only disappeared after at least 2 weeks [26]. Furthermore, it was investigated how and whether training the minipigs affects HRV during blood collection.

In summary, this experimental setup included comparison of blood samples taken from minipigs with conventional fixation or after clicker training in order to tolerate blood sampling without fixation. In addition, measurements of HR were performed, the effect of HRV depending on training was investigated, and salivary and serum cortisol levels were analysed before and during the blood collections.

## 2. Materials and Methods

### 2.1. Ethics Statement

The experiment was approved by the local authorities (State Office of Health and Social Affairs Berlin, approval ID G 0024/19) and conducted in accordance with the German Animal Welfare Act and the German Laboratory Animal Protection Ordinance as well as institutional guidelines.

### 2.2. Animals

The study was carried out with 12 female Ellegaard minipigs in a laboratory animal facility. The animals were, on average, 1.5 years old (SD 0.135) at the time of the experiment. The minipigs were kept individually or in small groups of up to three animals and always had eye contact with the minipigs in the opposite boxes. For training and blood sampling, the animals were separated individually in their familiar housing boxes. They did not need to fast for training or for blood collection. Prior to the experiment, no blood was taken from the pigs during regular husbandry. 

Blood sampling from the minipigs was scheduled for two consecutive days before and after the training phase. Ideally, sampling was to take place at the same time of day for all minipigs so that the circadian kinetics of cortisol would not result in major differences between the animals. For the same reason, the minipigs’ husbandry (housing, providing activities, feeding, and cleaning the boxes was standardised and remained unchanged during the training period.

### 2.3. Schedule and Training

At the beginning of the experiment, blood was collected from all animals in the conventional manner using a maxillary sling for fixation. The maxillary sling consisted of a rubberised steel cable. This was followed by a 12-day period of clicker training. At the end of the training phase, blood collection was repeated using the trained method without physical restraint.

The training of the minipigs included a total of 12 training days, during which the animals underwent two to four training sessions per day. The duration of the training sessions ranged from two to four minutes. Each session was started and ended by the acoustic start signal from a stopwatch, which was recognisable to the animals. The training method was clicker training. Animals were first classically conditioned to the clicker sound as the promise of an immediately following food reward (small apple pieces). This was followed by operant conditioning with increasing challenges [27,28]. A detailed training plan was created for this purpose. The animals were first conditioned to touch an object, a so-called target, with the proboscis disk and finally to remain with their snout in the target without moving for up to a minute. During this time, they were trained to allow a person initially to touch the neck and finally to insert a cannula and collect blood from the cranial vena cava without fixation. All minipigs were very motivated during training and were often excited at the beginning of training in anticipation of the reward.

**Figure 1:**
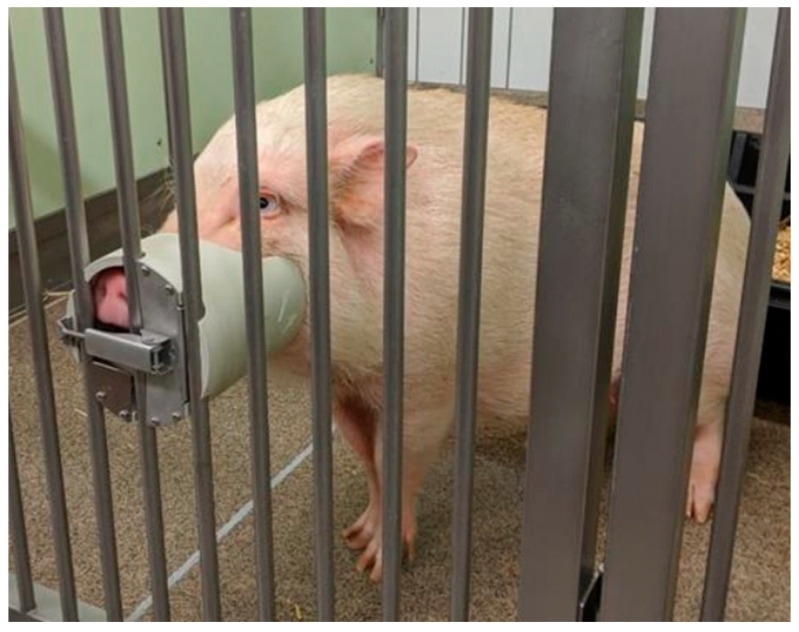
Minipig standing with the head held in the plastic target tube.

A plastic tube adjusted to the pig’s snout was used as a target and could be attached to the gage grid of the door of the enclosure (Figure 1). Since the mounting height can be individually adjusted to the size of the animal, it supports the desired positioning of the animal’s head and at the same time allows the pig to be rewarded through the tube during the desired action without leaving the target. To reward the animals after the clicker signal, small pieces of apple were identified as most attractive to the animals. The rewards were offered to the animals through the rear part of the target tube with the help of tongs.

### 2.4. Sample Collection, Sample Preparation, Heart Rate Measurement

The stress that the animals experienced during the blood collection was measured and compared using determinations of cortisol concentrations in saliva samples that were taken before and after the blood collection, as well as in the blood (serum) samples that were collected as part of the experiment.

Saliva samples were collected before both blood collections at rest (T0) and again 10 (T1), 30 (T2), and 50 min (T3) after blood collection. The saliva samples were taken from the side of the mouth using a Salivette (Salivette Cortisol^®^, SARSTEDT AG & Co., Nuembrecht, Germany), centrifuged (1000× *g* for 2 min), and then frozen at −20 °C until measurement. When blood was taken from the minipigs before the training phase, the minipigs were first fixed using an upper jaw sling, and the head was pulled upwards. A 2–3 mL volume of blood was then taken from the cranial vena cava using a sterile disposable cannula (20 G × 2 4/5 0.9 × 70 mm, Sterican, B. Braun Melsungen AG, Melsungen, Germany) and Sarstedt Monovette^®^, SARSTEDT AG & Co., Nuembrecht, Germany). The person who took the blood sample knelt diagonally to the right in front of the minipig and had free access to the animal’s neck. The maxillary sling was removed from the animal’s upper jaw after the cannula was removed. Similar to the saliva samples, the blood samples were cooled on ice immediately after collection and centrifuged on the same day at 2000× *g* for 10 min (Thermo Scientific Heraeus^®^ Labofuge^®^ 400R centrifuge (Thermo Scientific, Altrincham, UK)).

**Figure 2:**
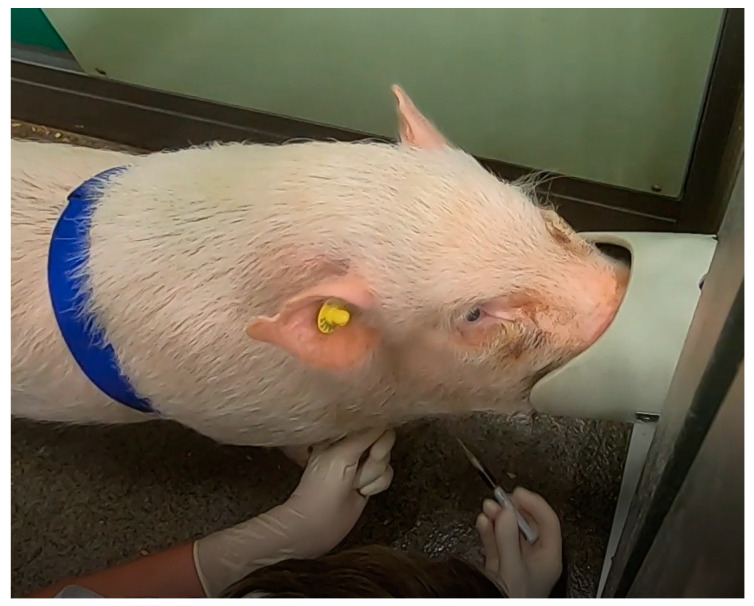
Blood collection from a trained, unrestrained minipig.

Blood sample collection from the untrained and the trained animals only differed in the fixation. The trained animals stood freely with their noses in the target tubes during blood collection (Figure 2).

To determine the cortisol concentration in the saliva and serum samples, competitive enzyme-linked immunosorbent assays (ELISAs) were used (Cortisol Salivary ELISA and Cortisol ELISA for serum and plasma; IBL International, Hamburg, Germany). The cortisol ELISAs employed have already been validated for use in pigs [29]. A Tecan Sunrise™ microtiter plate reader with the associated software Magellan™ version 7.2 from Tecan (Tecan Trading AG, Maennerdorf, Switzerland) was used for the measurements. The measurement units for serum and saliva cortisol were ng/mL.

The IBM^®^ SPSS^®^ Statistics program (IBM^®^, New York City, USA) and Microsoft^®^ Excel (Microsoft^®^ Corporation, Washington, DC, USA) were used for statistical analysis.

The HR was measured before and during blood samples to assess stress. 

HRs were measured for each minipig for 5 min at rest (A) and 5 min during blood collection ((B); 2.5 min before to 2.5 min after blood collection). The means of these 5 min measurements from the trained and untrained animals were compared in a paired-sample *t*-test, and the standard deviation, standard error, and *p*-value were determined. 

**Figure 3:**
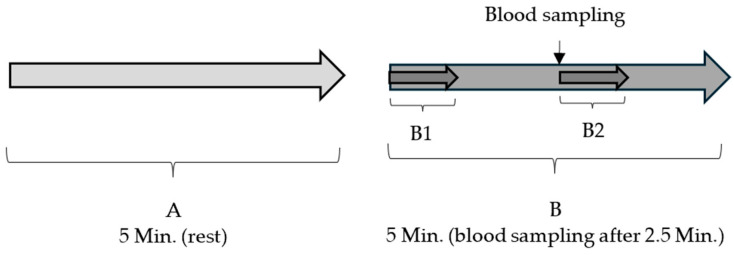
Illustration of the sections of HR measurements for statistical analysis. The HR of twelve minipigs was measured before the blood sampling (rest) for 5 min (**A**) and during the blood sampling for 5 min (**B**). Two 60 s sections (B1 and B2) of HR measurement during blood sampling were compared with each other in order to better represent the changes within the five-minute measurement period.

In addition, the five-minute measurement during blood sampling (B) was divided into different sections to better illustrate the fluctuations within the measurement period. The first section (B1) was the first 60 s of the measurement period (i.e., 2.5 min before the cannula was inserted). The next section (B2) was 60 s from the cannula puncture in the neck (Figure 3).

The means of this sections were then subjected to a paired-sample *t*-test, first for the untrained minipigs and then for the trained minipigs. A paired-sample *t*-test was also carried out on the second measurement section (B2) of the untrained and trained animals. The standard deviation and standard error were determined for all measurements.

For the measurements, Polar H10 Sensors^®^ (Polar Electro Oy, Kempele, Finland) and measuring straps were used; they were attached around the animals’ thorax caudal to the left elbow. These have already proven successful in other studies for measuring HR in pigs [13,14]. The skin of the minipigs at the level of the heart on the left chest wall, the position of the measuring sensor, as well as the measuring sensor itself were moistened with lubricant (WDT Gleitcreme Bengen^®^, WDT, Garbsen, Germany) to ensure optimal contact and the most complete recording possible. The measurements were controlled via a mobile phone using the Polar apps (Polar Beat^®^ and Polar Flow^®^ (Polar Electro Oy, Kempele, Finland)), saved, and later exported to Excel tables.

### 2.5. Statistical Analysis

The work investigated the effect of training on 12 minipigs. There was no separate control group; they provided their own control. The *p*-value accepted for the study was 0.05.

The salivary cortisol measurements were first subjected to an analysis of variance (ANOVA) for repeated measurements. In this case, the measurement repetition has 4 levels: The resting measurement (T1) and the measurements 10 min (T2), 30 min (T3), and 50 min (T4) after blood sampling. The between-subjects factors were classified as “trained” and “untrained”.

Mauchly’s test of sphericity was used, and a correction method was applied. 

The Greenhouse–Geisser test was chosen to determine the significance of the differences between the various represented stages. Using the within-subjects contrast test, the changes between each two successive stages among the four (between stages 1 and 2, stages 2 and 3, and stages 3 and 4) and the significance were determined. In addition, such an interaction was assessed between the within-subjects factors with the different measurement times examined. 

The test of between-subjects effects represents the difference between the two groups (trained and untrained). Using the parameter estimates, the two groups were assigned to each of the 4 measurement points compared, and the significances were calculated. The residuals for each of the four time points were checked and showed that the data followed a normal distribution.

The means of the HR measurements (A and B) were subjected to a paired-sample *t*-test. A paired-sample *t*-test was also carried out with the second measurement section (B2) of the untrained and trained animals, and another paired-sample *t*-test with measurements B1 and B2. The standard deviation and standard error were determined for all measurements.

Measurements concentrated on short-term fluctuation induced by the aversive stimulus due to the procedure of blood collection in minipigs. In order to determine the heart rate variability, heart rates were estimated for each minipig in the untrained and trained states. Maximum and minimum values of each minipig were compared during measurement A as well as during measurement B. As described before, a period of 5 min HR measurement was used for evaluation according to current recommendations (von Borell et al. 2007 [21]). The difference was calculated from the delta value of the measurements A and B. These were then compared for all animals in the paired-samples *t*-test, and the standard deviation and standard error, as well as the significance, were calculated.

## 3. Results 

### 3.1. Salivary Cortisol

The test of between-subjects effects of the ANOVA analysis of variance showed a significant difference between the two groups “trained” and “untrained” with a *p*-value < 0.001. When looking at the parameter estimates, no difference (*p*-value of 0.26) between the cortisol values at time T0 between the trained and untrained animals could be observed.

**Figure 4:**
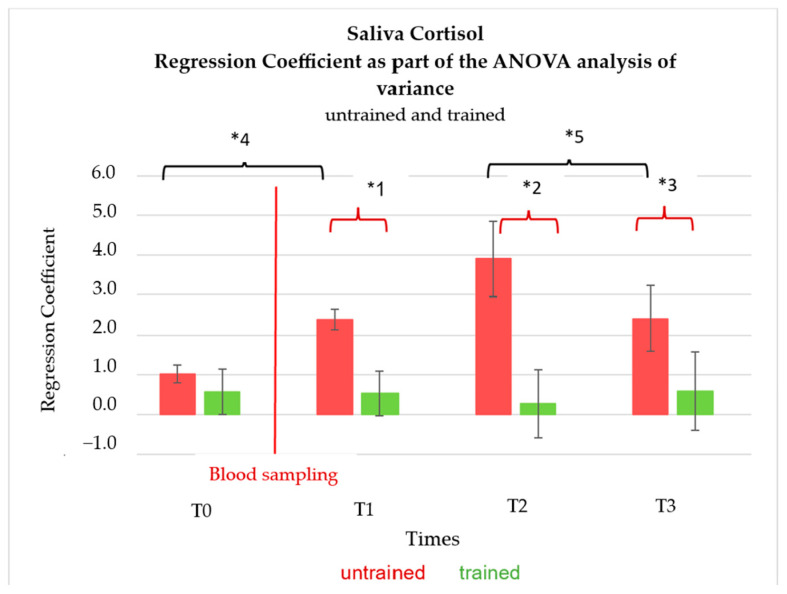
ANOVA analysis of variance with 4 levels; n = 12. Comparison of regression coefficients of salivary cortisol values untrained (red bars) and trained (green bars) at rest (T0), as well as 10 (T1), 30 (T2), and 50 min (T3) after blood collection (red line). Error bars: 95% confidence interval. Significances: *1 *p* = 0.001, *2 *p* = <0.001, *3 *p* = 0.013, *4 = 0.006, *5 = 0.006.

The cortisol values of the untrained animals at T1, T2, and T3 were higher than those of the trained animals and the differences are significant with a *p*-value of <0.001 (at T1 and T2) and a *p*-value of 0.013 (T3) (Figure 4). The cortisol levels in the saliva of the untrained animals at T1, T2, and T3 were also significantly increased compared to time T0 (*p*-value = 0.006 (Table 1)).

### 3.2. Serum Cortisol

Values of 10 out of 12 animals were included in the evaluations because it was not possible to obtain the required amount of blood for analysis from two animals. No significant difference (t (9) = 2.052, *p* = 0.07) was observed in serum cortisol concentrations between the untrained and trained minipigs (Table 2).

### 3.3. Heart Rate Frequency and Heart Rate Variability

**Figure 5:**
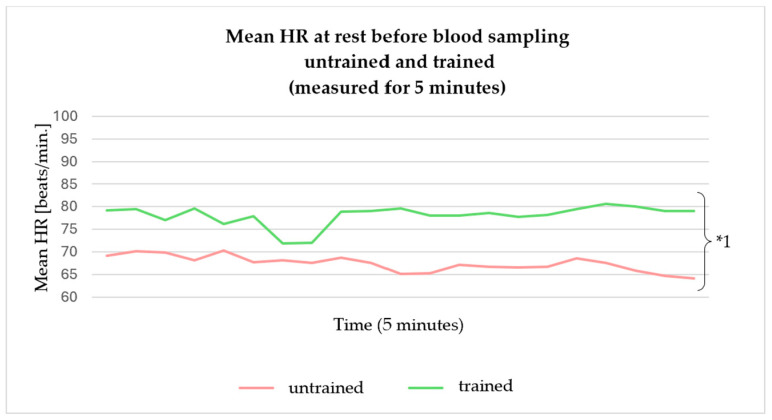
Mean HRs of the 12 minipigs in untrained and trained states, measured over 5 min measured at rest before the blood sampling. The untrained state is shown in red, the trained state in green. Significances: *1 mean HR untrained vs. trained animals *p* = 0.023.

The mean HR in measurement period A was significantly higher in the trained minipigs than in the untrained minipigs (t (11) = −2.64, *p* = 0.023). The trained animals showed a higher HR right at the start of the measurement, which remained essentially unchanged during the five-minute measurement period (Figure 5).

**Figure 6:**
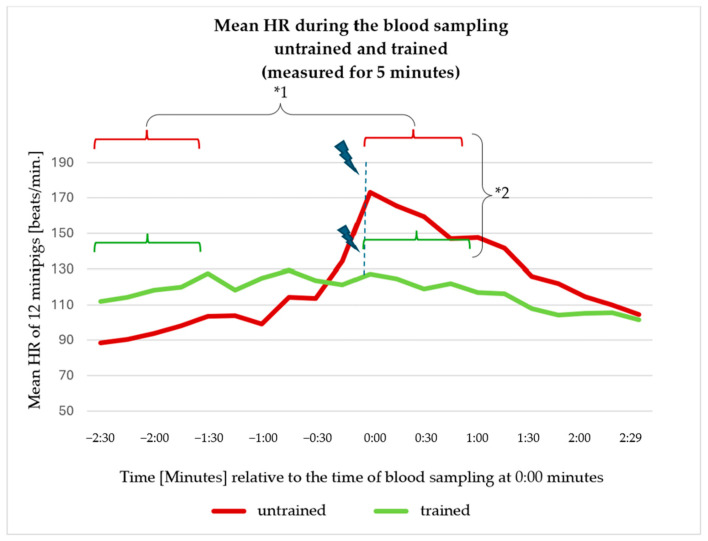
Mean HRs of the 12 minipigs in untrained and trained states, measured over 5 min around the time of blood sampling. The untrained state is shown in red, the trained state in green. The time of blood collection (blue dotted line and blue thunder symbols) was defined as 0:00. The application of the maxillary sling took place within 20−30 s before the neck was pierced with the cannula. Significances: *1 B1 vs. B2 in untrained animals *p* = <0.001; *2 untrained vs. trained animals at period B2 *p* = 0.001.

Looking at the mean HRs during measurement period B, it is immediately noticeable that the HR of the untrained minipigs increased sharply at the time of blood collection and then fell again until the end of the measurement (Figure 6). Although the HR of the trained minipigs was slightly higher at the beginning of the measurement than that of the untrained animals over the same period, it did not show a significant increase around the time the blood is taken. Statistically, no significant difference could be determined between the trained and untrained animals when comparing measurement period B (t (11) = 0.459, *p* = 0.655).

Comparing the smaller measurement sections of 60 s, B1 and B2, to show the changes in HR within the measurement period B, it can be seen that the untrained animals had a significantly higher HR within period B2 than the trained animals (t (11) = 4.678, *p* = 0.001). Comparing B1 and B2, there was a significant increase in the untrained minipigs (t (11) = 6.021, *p* = <0.001). There was no significant difference in the trained animals when comparing the same measurement periods (t (11) = 0.375, *p* = 0.715) (Table 3).

When comparing the HRV, there was a significant difference (*p* = 0.003) between the untrained and trained minipigs t (11) = 3.704, *p* = 0.003. The HRV of the untrained minipigs was more than twice as high as that of the trained minipigs. This means that the HR of the untrained minipigs showed significantly higher fluctuations than that of the trained minipigs.

## 4. Discussion 

The results show that blood sampling from pigs restrained using a maxillary sling leads to stress, demonstrated by increased cortisol concentrations in saliva and by increased HR and decreased HRV after the blood sampling. These results match the results of other studies [9,30]. In both studies, pigs were restrained with a maxillary sling for 5 min (without blood sampling), and there was a significant increase in salivary (and serum) cortisol levels.

The results of the measurements after training show that there is no significant increase in HR during blood sampling and that salivary cortisol levels do not increase either if the animals are not restrained during blood collection. Both groups experienced puncture with the cannula through the skin and deeper layers of tissue on the neck up to the cranial vena cava and any necessary corrections to the cannula position to the same extent. Nevertheless, there was neither an increase in salivary cortisol nor a significant change in HR associated with the puncture in the trained, non-restrained animals. This suggests that the major amount of the stress observed in the untrained animals could be reduced by training, desensitisation (caused by training), and avoiding fixation with the maxillary sling. The present work shows that minipigs can be trained to tolerate blood sampling without restraint. Blood could be taken from 10 of 12 animals after training without restraining them. Two animals initially tolerated the puncture with the cannula but were overall too restless, such that corrective movements with the cannula would have posed too high a risk of injury to the animal, and the blood collection was stopped. The good training success of the other animals suggests that with a longer training period, blood sampling would also have been possible from these two animals. The willingness to train was very high among all animals. Some animals were even so over-motivated that the training had to be briefly interrupted so that the animals could calm down again and be trained in a focused manner.

Taking blood from unrestrained pigs requires intensive continuous training in order to achieve reliable tolerance from the animals. If this is missing, the lack of fixation poses a higher risk of injury to the animal and the sample taker. In general, there is a risk of injury to the phrenic nerve when taking blood via the cranial vena cava [31]. There are alternative suggestions in the literature to reduce this risk, such as puncturing the cephalic vein or taking small amounts of blood from the auricular vein [32,33]; whether this method is also an option for standing animals could be investigated in further studies. On the other hand, blood collection from a successfully trained minipig without fixation is also gentler for the sample taker because less effort is needed. In addition, the noise pollution is significantly lower. Refinement means reducing the burden but also refining the methods.

Another aim of the study was to show whether training can reduce stress during blood sampling. 

Both stress parameters, salivary cortisol and HR, could be significantly reduced through positive reinforcement training. Other studies have also shown that training animals can minimise their stress level [34,35]. The HR was significantly higher and the HRV significantly lower in the untrained animals during blood collection than in the trained animals. However, the HR at rest and at the beginning of the 5 min measurement phase during blood sampling was slightly higher in the trained animals than in the untrained animals. This can be explained by excitement caused by the anticipation of the training sessions every morning and the expectation of rewards. A similar observation was reported by Zebunke, Puppe, and Langbein [36]. In another study, pigs reacted to the sound of the feed cart being pushed into the barn with an increase in HR in anticipation of feeding [37]. This may also have contributed to the non-demonstrable difference in serum cortisol concentrations between the two groups.

When comparing the serum cortisol values, it was shown that despite the lack of significance in the group comparison, the individual animals that had high serum cortisol levels before training particularly benefited from the training and showed lower serum cortisol values after the training. By forgoing a separate control group, it was possible to determine such intra-individual changes, and the number of animals in the experiment was kept as low as possible in accordance with the 3R principle (reduction).

This training- and time-intensive method is particularly suitable for animals that are kept for a sufficient period of time to enable adequate training time and maintenance of training success. Although the training requires an increased amount of work at the start of the study, time can be saved as the study progresses once the animals have been trained. Another advantage of the training is that it provides enrichment for the animals and, in addition to activity, it is also a cognitive challenge that can increase animal welfare [15,38]. Negative interactions with humans can lead to increased stress and fear towards humans [39,40]. Training therefore reduces fearful behaviour in animals [15,41] and gives animals control and the ability to make decisions, for example, on whether to participate voluntarily in a training session or not [42]. By starting and ending the training sessions with a sound signal, the minipigs were taught by conditioning that training follows the signal and that manipulation only takes place after the signal. This led to better predictability for the animals because after a while they knew what action would follow the sound signal, and they might also anticipate that if the signal did not appear, nothing would happen. Predictability contributes to animal wellbeing [43] and therefore represents a refinement aspect.

The closer contact can improve the human–animal relationship [17]. In general, training promotes the relationship between humans (or, more, specifically, trainers) and animals, creates trust, and can not only provide entertainment and enrichment for the animal but also improve working conditions and job satisfaction for people if, thanks to training, fewer coercive measures are needed.

Training animals also brings challenges such as finding the right reinforcer. In the present work, apples were used, which were very suitable as reinforcers for all minipigs used in the experiment. To avoid hand conditioning, the apple pieces were passed to the minipigs using tongs. Another aspect that training brings with it is the individually different training progress. There are inter-individual differences in the nature and personality of animals [44], some of which require individual adaptation of the training plan. For some animals, training sessions had to be prolonged or repeated to support the training success, while for some other, hypermotivated animals, trainings needed to be interrupted and restarted after some time. If several trainers are involved in the training, it is also essential that they coordinate their actions precisely so as not to confuse the animals and impede the success of the training. Training in group housing is not impossible, but it is certainly much more challenging and time-consuming than if the animals can be separated for individual training.

The implementation of intensive training and cognitive enrichment in animal husbandry has gained attention in recent years, particularly in zoos and experimental settings. These approaches, rooted in operant conditioning and behaviour analysis, aim at improving animal welfare by providing cognitive challenges and promoting naturalistic behaviours [45]. Such practices can positively affect animal behaviour, enhance environmental predictability, and activate reward-related brain systems [46]. However, their application in agricultural settings remains limited due to the focus on intensive production systems [47]. The stress experienced by farm animals in these environments is not merely a reflex reaction but is influenced by psychological factors and individual perception [48]. While progress has been made in zoo animal welfare through training and enrichment, further research is needed to address the challenges of multiple chronic stressors in intensive farming and to develop suitable physiological criteria for assessing long-term adaptive changes [48].

The initially very high investment of time required may represent a limitation for its use, and not only in conventional animal husbandry. However, here too, the results of the study can be seen as an incentive to optimise repetitive interventions, treatments, or breeding measures and make them less stressful. There remains room for further studies to quantify distress in animals and to develop and validate methods to minimise distress as well as for studies on cost effectiveness. The results allow the conclusion that the major part of stress during blood collection in minipigs using the conventional blood collection method is caused by fixation using a maxillary sling. The stress can be significantly reduced through training. At the same time, the training represents cognitive enrichment for the animals [36,44] and thus fulfils an additional aspect of the 3R principle as applied refinement, all the more so as a lack of stimulation can lead to undesirable behaviour [49,50]. Improvement of experimental results may also be contributed to by training, as fewer distortions of results due to stress and therefore lower standard deviations may be achievable, possibly reducing the number of animals required for an experiment. This meets the aspect of reduction and may also contribute to better science [51]. 

In general, a strong connection between animals and animal caretakers or scientific staff and a deeply felt commitment to improving animal welfare can positively advance a research institution’s culture of care.

## 5. Conclusions 

Taking blood employing fixation in a maxillary sling is stressful for minipigs and leads to an increase in cortisol in saliva, as well as an increase in HR and a decrease in HRV. The stress is essentially caused by fixation in a maxillary sling. Puncture with a cannula, compared to the fixation method, does not seem to contribute significantly to the stress on the animals. It is possible to train minipigs by positive reinforcement to have blood withdrawn without restraint. Training can reduce the stress that the minipigs experience during blood collection and improve animal–human interaction.

## Figures and Tables

**Table 1:** Mean, standard deviation, and standard error of the mean of the saliva cortisol concentrations from the untrained and trained minipigs.

Time of Saliva Sample Collection	Mean Saliva Cortisol (ng/mL)	Standard Deviation	Standard Error of the Mean
T0, untrained	1.03	1.30	0.37
T1, untrained	2.40	1.28	0.37
T2, untrained	3.90	2.00	0.58
T3, untrained	2.42	2.29	0.66
T0, trained	0.58	0.35	0.10
T1, trained	0.53	0.34	0.10
T2, trained	0.27	0.28	0.08
T3, trained	0.59	0.54	0.16

**Table 2:** Mean, standard deviation, standard error of the mean, and *p*-value of the serum cortisol measurements from the untrained and trained minipigs.

Serum Cortisol	Mean Serum Cortisol (ng/mL)	Standard Deviation	Standard Error of the Mean	*p*-Value
untrained	79.15	50.54	15.98	0.07
trained	45.01	10.65	3.37	

**Table 3:** Mean, standard deviation, standard error of the mean, and *p*-value of different sections of the HR measurements. Significant *p*-values are marked with bold letters. A = 5 min measurement at rest. B = 5 min measurement during blood collection, starting 2.5 min before insertion of the cannula. B1 = first 60 s of B. B2 = 60 s after insertion of the cannula in the neck of the minipig for blood collection.

Section of the HR Measurement	Mean HR (Beats/Minute)	Standard Deviation	Standard Error of the Mean	*p*-Value
B1, untrained	95	19.06	5.62	**<0.000**
B2, untrained	160	25.18	7.27	
B1, trained	118	17.10	4.94	0.243
B2, trained	123	17.29	4.99	
B2, untrained	160	25.18	7.27	**0.001**
B2, trained	123	17.29	4.99	
A, untrained	67	14.60	4.21	**0.023**
A, trained	79	8.86	2.56	
B, untrained	123	16.38	4.75	0.462
B, trained	118	15.03	4.34	

## Data Availability

The data presented in this study are available on request from the corresponding author as a part of her doctoral thesis.
